# The use of digital psychosocial intervention (DIALOG+) via a mental health community team at the University Clinic of Psychiatry - Skopje

**DOI:** 10.1192/j.eurpsy.2023.1846

**Published:** 2023-07-19

**Authors:** M. Milutinovic, S. Bajraktarov, A. Novotni, L. Novotni

**Affiliations:** University Clinic of Psychiatry - Skopje, Skopje, North Macedonia

## Abstract

**Introduction:**

The University Clinic of Skopje – Skopje was part of two Horizon 2020 projects – IMPULSE and RECOVER-e that finished in December 2021. The advances in the field of community mental health in the capital of Skopje, the idea to combine the best aspects of the aforementioned projects and the need of continual implementation and research on an evidence-based community-based service delivery model for recovery-oriented care led to this study which aims to improve functioning, quality of life, and mental health outcomes for people with severe and enduring mental ill health, such as schizophrenia, bipolar disorder, severe depression.

**Objectives:**

The objectives of the study are: - to design, implement and evaluate recovery-oriented care for people with severe mental illness in community settings using a psychosocial digital intervention - DIALOG+;- to recognize the value of experiential knowledge through inclusion of peer experts as members of community mental health teams;- to develop scale-up plans for national decision-makers, as informed by the intervention’s implementation and impact, for sustained implementation and scale up after the research study’s timeline.- to improve the conditions of people suffering from psychotic disorders and to overcome financial barriers encountered in the treatment of psychotic disorders in N. Macedonia.

**Methods:**

DIALOG+ represents an affordable and effective intervention which has already demonstrated positive outcomes in previous research. This study involves the use of the DIALOG+ intervention during home visits, so that patients themselves can decide which aspects of their life that they would like to discuss and work on improving. DIALOG+ lets them rate 12 domains that are related to quality of life, such as physical health, relationships and employment. Patient decide which of these they would like to discuss in detail during the meeting. There is then a 4-step approach to help improve this aspect of their life, using the principles of solution-focused therapy. Researchers will collect information about demographic characteristics, quality of life, and symptoms in patients taking part in the study through the administration of questionnaires and clinical scales.

**Results:**

The study is still in phase of completion. The results will be shown at the EPA Congress 2023.

**Conclusions:**

Having the previous positive outcomes from IMPULSE and RECOVER-e, with this combined approach we expect even more improvement in functioning and better quality of life in patients suffering from severe and enduring mental ill health.

**Disclosure of Interest:**

None Declared

